# Ultrasensitive Detection of Trace Silver Ions Using MoS_4_^2−^-Intercalated LDH Nanosheets Enhanced SPR Sensor

**DOI:** 10.3390/s24185973

**Published:** 2024-09-14

**Authors:** Linlong Tang, Guilian Lan, Penglei Ma, Yu Jia, Xiaojian Zhang, Peng Luo, Wei Wei

**Affiliations:** 1 Chongqing Key Laboratory of Multi-Scale Manufacturing Technology, Chongqing Institute of Green and Intelligent Technology, Chinese Academy of Sciences, Chongqing 400714, China; 2 College of Optoelectronic Engineering, Chongqing University, Chongqing 400044, China; 3 School of Optoelectronic Engineering, Chongqing University of Posts and Telecommunications, Chongqing 400065, China

**Keywords:** surface plasmon sensor, LDH nanosheets, silver ions

## Abstract

The widespread use of silver raises concerns about environmental and health risks, necessitating highly sensitive detection methods for trace silver ions (Ag^+^). Surface plasmon resonance (SPR) sensors offer benefits like label-free detection and rapid response, but their sensitivity for Ag^+^ detection is limited due to weak ion adsorption. Here, we developed an SPR sensor with MoS_4_^2−^-intercalated NiAl-layered double hydroxide (LDH) as the adsorption layer of Ag^+^ to enhance detection sensitivity. Our sensor achieves a sensitivity of 254.75 nm/μg/L and detects Ag^+^ at a low concentration of 2.8 pM, outperforming various existing sensors. It also shows excellent repeatability, long-term stability, and selectivity, proving effective in real-world environmental samples. This work advances high-performance SPR sensors for heavy metal ion detection.

## 1. Introduction

Heavy metal accumulation in biological systems causes irreversible and permanent damage to human health and the ecosystem, posing a significant global public health and safety concern [1,2,3]. Silver, a common heavy metal, is extensively utilized in various industries including jewelry, catalysts, medicine, and solar panels due to its strong inhibitory and bactericidal properties. These applications result in an annual release of approximately 2500 tons of silver into the environment, exacerbating environmental pollution and health risks [4,5]. Consequently, there is urgent need for selective and sensitive monitoring of trace levels of silver ions. Traditional detection methods, including fluorescence, colorimetry and electrochemistry, each offer distinct advantages [6,7,8]. For instance, fluorescence provides high sensitivity and enables the detection of low concentrations, while colorimetry is straightforward and cost-effective for field applications. However, these techniques often require complex sample preparation, are prone to interference from other substances, and may have limited dynamic ranges or require careful calibration [9,10,11]. In this context, surface plasmon resonance (SPR) spectroscopy [12,13,14] presents a promising alternative for detecting heavy metal ions, offering rapid response times, non-destructiveness, and high reproducibility. Notably, SPR sensors can monitor unlabeled targets in real-time near the sensing surface, making them particularly suitable for heavy metal ion detection. However, the sensitivity of conventional SPR sensors for low-concentration ion solutions remains a challenge due to limitations in ion adsorption, hindering further performance improvements and practical applications.

To overcome this limitation, researchers have extensively explored the modification of the sensor surface with ion-adsorption membranes [15,16,17,18,19,20,21,22,23,24]. This strategy amplifies the weak local refractive index change enabled by ion binding, thus enhancing the SPR signal. Organic materials with functional groups, such as chitosan and polyacrylic acid [25], have been commonly employed for surface modification to improve the adsorption capacity of the sensor, leading to detection limit improvements from mg/L to μg/L. However, achieving further sensitivity improvements remains challenging due to issues like poor chemical stability, relatively low specific surface area, and weak mechanical properties. Recently, two-dimensional (2D) inorganic materials, such as MoS_2_, MoSe_2_ and MXene [19,20,21,22,23], have emerged as promising alternatives for SPR sensor surface modification due to their well-defined pore structures and superior surface properties, leading to enhanced sensitivity compared to their organic counterparts. Among these materials, 2D layered double hydroxides (LDHs) have attracted considerable attention due to their large specific surface area, excellent chemical stability, and high anion exchange capacity [26,27,28]. Importantly, the layered structure of 2D LDHs allows for controlled interlayer functional groups, facilitating the adsorption of anions and, consequently, the capture of heavy metal ions. Given these advantages, investigating the integration of 2D LDHs into SPR sensors holds significant promise for enabling highly selective and sensitive detection of Ag ions.

This study presents a new label-free SPR sensor for Ag^+^ detection with enhanced sensitivity and selectivity. The sensor utilizes MoS_4_^2−^-intercalated NiAl-LDH nanosheets as the adsorption layer, in which MoS_4_^2−^ intercalation replaces the original CO_3_^2−^ anion within the LDH structure, resulting in a material with high adsorption capacity and good selectivity towards Ag^+^. The performance of the sensor was optimized by controlling the synthesis process of the MoS_4_-LDH nanosheets and the spray volume applied to the sensor surface, which influences the layer thickness. This optimization yielded a remarkable sensitivity of 254.75 nm/μg/L for Ag^+^. Further analysis based on the Langmuir adsorption model revealed an exceptionally low detection limit of 2.8 pM, surpassing various reported Ag^+^ sensors based on diverse sensing principles. Additionally, the proposed sensor demonstrates excellent repeatability, long-term stability, good selectivity for Ag^+^, and effective performance in real-world environmental samples. This approach holds significant promise for highly sensitive Ag^+^ detection and expands the utility of SPR sensors in metal ion sensing applications.

## 2. Structure and Principle of the LDH-Based SPR Sensor

**Figure 1:**
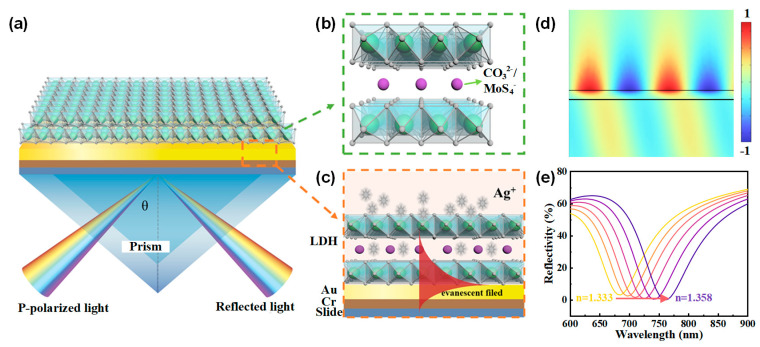
(**a**) Schematic configuration of the proposed SPR sensor utilizing NiAl-LDH nanosheets as the adsorption layer. (**b**) Illustration of the layered structure of NiAl-LDH, featuring alternating positively charged metal hydroxide layers and negatively charged interlayer anions. (**c**) Working principle of the NiAl-LDH-based SPR sensor. (**d**) Normalized electric field distribution of surface plasmons excited at an incident angle of 73°. (**e**) Resonant shifts to longer wavelengths as the refractive index of the medium on the surface of Au increases.

The SPR sensor chips were prepared on BK7 glass substrates by depositing a 5 nm chromium adhesion layer followed by a 50 nm gold film using vacuum evaporation. A Kretschmann configuration SPR system was employed, and Figure 1a, c depict the apparatus and cross section of the SPR sensor. Figure 1b illustrates the schematic structure of NiAl-LDH laminates, featuring positively charged layers and interlayer anions connected by non-covalent bonds. These layers consist of NiO_6_ octahedra with isomorphous substitution, where some divalent cations (Ni^2+^) are replaced by trivalent cations (e.g., Al^3+^). The interlayer anions typically include CO_3_^2−^, NO_3_^−^, MoS_4_^2−^, etc., which ensure the LDHs remain electrically neutral overall. Additionally, the interlayer space may accommodate water molecules. LDHs are generally expressed by the formula [M_1−x_^2+^ M_x_^3+^(OH)_2_]^x+^[(A^n−^)_x/n_∙mH_2_O]^x−^, where M^2+^ and M^3+^ represent divalent and trivalent cations; x is the molar ratio of M^3+^/(M^2+^ + M^3+^), typically ranging between 0.17 and 0.33; and A^n−^ is the interlayer anion.

When transverse magnetic (TM) polarized light is directed at a specific incident angle (73° in our study) onto the BK7 prism, surface plasmons (Figure 1d) can be excited on the gold film by the evanescent wave, resulting in a dip in the reflection spectrum at the resonant wavelength. As the refractive index of the medium on the surface of the gold film changes, while keeping the incident angle fixed, the resonant wavelength shifts to maintain the resonant conditions of the surface plasmons, as illustrated in Figure 1e. The NiAl-LDH laminates can selectively adsorb Ag^+^ ions, which may enhance the shift of the resonant wavelength upon Ag^+^ adsorption, thereby improving the sensitivity of the SPR sensor.

## 3. Material Preparation and Characterization

### 3.1. Reagents and Instruments

Two-dimensional CO_3_-LDH nanosheets with an average diameter of 30 nm were purchased from XF NANO Co., Ltd. (Nanjing, China). Ammonium tetrathiomolybdate ((NH_4_)_2_MoS_4_) was obtained from Sigma Aldrich. Intercalation of MoS_4_^2−^ anions was achieved by combining CO_3_-LDH with (NH_4_)_2_ MoS_4_ in deionized water and stirring for 9 h to obtain the MoS_4_-LDH nanosheets. Standard solutions of heavy metal ions (Ag^+^, Pb^2^^+^, Cr^2^^+^, and Hg^2^^+^) at a concentration of 20 μg/L were purchased from Wan Jia Co., Ltd. (Beijing, China). These solutions were independently diluted with deionized water to prepare various concentrations for further experiments. The refractive indices of all solutions were measured using an Abbe refractometer with a resolution of 0.001 and found to be similar.

The structural properties of the samples were examined using transmission electron microscopy (JEOL JEM-3100F field emission TEM, JEOL Ltd., Tokyo, Japan). The morphology and elemental composition of the LDH nanosheet films were characterized by field emission scanning electron microscopy (JEOL JSM-7800F, JEOL Ltd., Tokyo, Japan) and energy dispersive spectroscopy (EDS) (FEI Nova 400, Thermo Fisher Scientific, Hillsboro, OR, USA, operated at 10 kV). Fourier transform infrared (FTIR) spectra were recorded using an FTIR spectrometer from PerkinElmer (Spotlight 400, Waltham, MA, USA). Additionally, the phase composition of the prepared samples was analyzed by X-ray Diffraction (XRD, Bruker D8 Discover, Bruker Corporation, Billerica, MA, USA), while the chemical environment and surface compositions were investigated using X-ray photoelectron spectroscopy (XPS, Kratos AXIS Ultra, Kratos Analytical, Manchester, UK).

### 3.2. Characterization of the LDH Films

**Figure 2:**
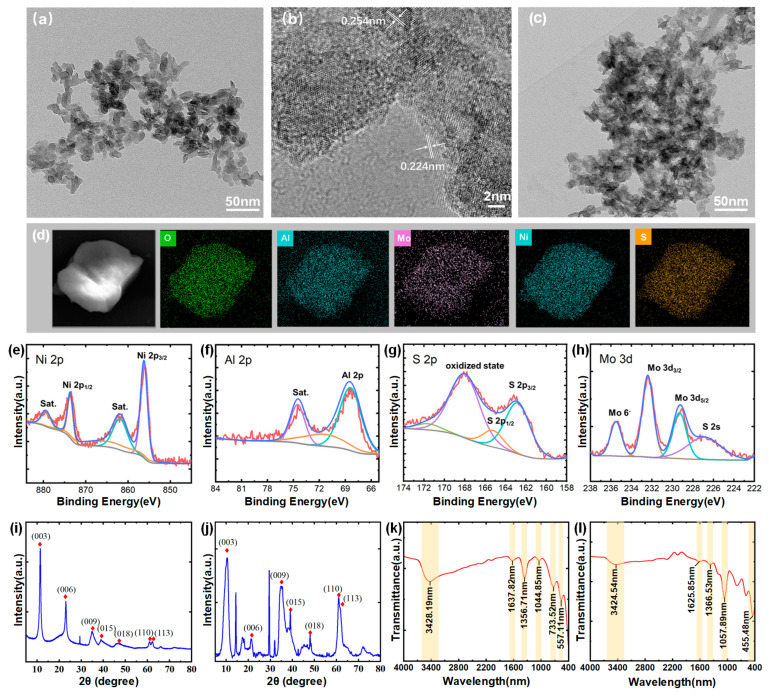
(**a**) TEM image and (**b**) HRTEM image of CO_3_-LDH nanosheets. (**c**) TEM image of MoS_4_-LDH nanosheets film. (**d**) Elements mapping images of MoS_4_-LDH. (**e**–**h**) Elemental composition of MoS_4_-LDH through XPS analysis. (**i**,**j**) XRD spectra of CO_3_-LDH and MoS_4_-LDH, respectively. (**k**,**l**) FTIR spectra of CO_3_-LDH and MoS_4_-LDH, respectively.

To characterize the structural properties of the LDH nanosheets, a TEM image at a scale of 50 nm for CO_3_-LDH is presented in Figure 2a. The average size of the nanosheets is approximately 30 nm, and they exhibit a two-dimensional layered morphology. In Figure 2b, high-resolution TEM (HRTEM) analysis of a single sheet confirmed its crystalline nature and identified lattice spacings of 0.254 nm and 0.224 nm, corresponding to the (009) and (010) planes of LDH crystals, respectively [29]. Figure 2c shows the TEM image of MoS_4_-LDH, there is no obvious difference between them and the CO_3_-LDH nanosheets. Figure 2d presents elemental mapping images of the prepared MoS_4_-LDH nanosheets, highlighting the distribution of Al, Ni, Mo, S, and O elements and confirming the expected elemental composition of the MoS_4_-LDH nanosheets.

X-ray photoelectron spectroscopy (XPS) analysis was further conducted to investigate the chemical states and elemental composition of the MoS_4_-LDH nanosheets. The corresponding spectra are presented in Figure 2e–h. The Ni 2p spectrum exhibits two main peaks at 873.6 and 855.7 eV, which can be assigned to Ni^2^^+^. The Al 2p peak at 68.5 eV confirms the presence of Al^3^^+^. The S 2p spectrum shows two peaks for MoS_4_-LDH at 168.6 and 164.0 eV, corresponding to polysulfide and surface sulfate, respectively. In the Mo 3d spectrum, the peaks at 235.1 and 232.4 eV correspond to Mo⁶^+^ (Mo 3d_5/2_ and Mo 3d_3/2_), while the peaks at 229.6 and 227.3 eV can be attributed to Mo^3^^+^ in Mo-S species (Mo 3d_5/2_). These XPS results confirm the successful preparation of MoS_4_-LDH nanosheets [30].

The crystal structures of CO_3_-LDH and MoS_4_-LDH nanosheets were determined via XRD analysis, illustrated in Figure 2i,j. Both figures exhibit diffraction peaks at 11.2°, 22.8°, 34.5°, 60.0°, and 61.3°, corresponding to the (003), (006), (009), (015), and (110) planes of LDHs (JCPDS No. 15-0087) [31]. Additional peaks in the MoS_4_-LDH spectrum suggest slight variations in the crystal structure of the nanosheets. Figure 2k presents the FTIR spectrum of CO_3_-LDH nanosheets. The observed peaks correspond primarily to vibrations of the metal hydroxide layers, interlayer anions, and adsorbed water, consistent with previously reported intercalation structures [32]. A broad peak at 3428 cm^−1^ originates from overlapping O-H stretching vibrations of both surface and interlayer water molecules. Peaks at 1356 cm^−1^ and 733 cm^−1^ correspond to the stretching and bending vibrations of interlayer CO_3_^2−^ anions, respectively. The peak at 1637 cm^−1^ indicates water molecule bending vibrations. Figure 2l shows the FTIR spectra of MoS_4_-LDH nanosheets, where the reduced intensity of the CO_3_^2-^ band at 1366 cm^−1^ and appearance of the Mo-S band at 455 cm^−1^ confirm CO_3_^2−^ ion exchange with MoS_4_^2−^ ions [30].

**Figure 3:**
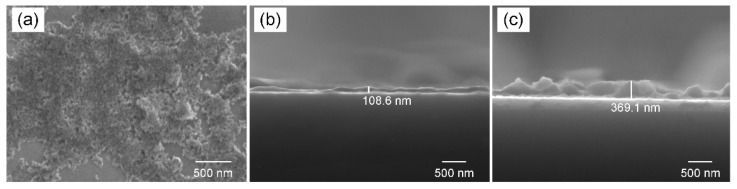
(**a**) Top-view SEM image of the deposited MoS_4_-LDH film. (**b**,**c**) Side-view SEM images of the deposited film with MoS_4_-LDH spray volumes of 100 and 300 μL, respectively.

To characterize the morphology and thickness of MoS_4_-LDH nanosheets, solutions with varying volumes were sprayed onto the substrates. The top-view SEM image in Figure 3a reveals a relatively continuous film at the micrometer scale. Figure 3b,c provide typical side-view SEM images of the deposited layers for MoS_4_-LDH spray volumes of 100 and 300 μL, respectively, at 2.5 mg/mL. The observed increase in layer thickness with the volume of MoS_4_-LDH sprayed suggests a direct correlation between the coating thickness and the amount of material applied. The sensing performance of MoS_4_-LDH-based SPR sensors, potentially affected by the thickness of the coating layer, will be further investigated in subsequent sections.

## 4. Performance of the MoS_4_-LDH-Based SPR Sensors

**Figure 4:**
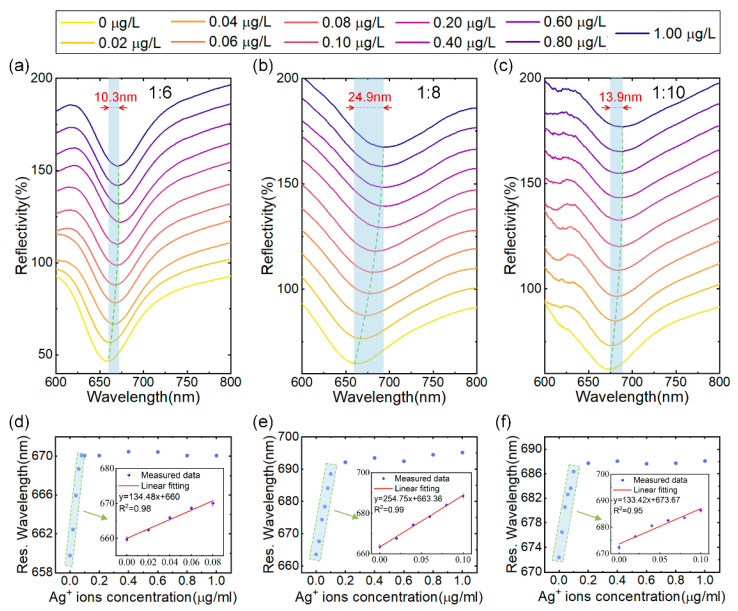
(**a**–**c**) Reflectance spectra of SPR sensors coated with MoS_4_-LDH at precursor ratios ((NH_4_)_2_MoS_4_ to CO_3_-LDH) of 1:6, 1:8, and 1:10, respectively, in response to varying Ag^+^ concentrations. (**d**–**f**) Extracted resonant wavelength shifts as a function of Ag^+^ concentrations from (**a**–**c**), with insets showing the linear range fitting.

The initial investigation focused on determining the optimal (NH_4_)_2_MoS_4_ to CO_3_-LDH ratio for SPR sensor performance during the synthesis of MoS_4_-LDH. MoS_4_-LDH dispersions were prepared at mass ratios of 1:6, 1:8, and 1:10, each at a concentration of 2.5 mg/mL. Utilizing a fixed spraying volume of 300 μL, three SPR sensors modified with MoS_4_-LDH at varying mass ratios were fabricated via the spraying method for Ag^+^ ion detection. The tested Ag^+^ solution concentrations ranged from 0.02 to 1.0 μg/L. The reflection spectra of the SPR sensors, depicted in Figure 4a–c, demonstrate that as the Ag^+^ solution concentration increases, the resonance wavelength of the reflection spectrum shifts towards longer wavelengths and eventually saturates. The maximum wavelength shifts corresponding to saturation are 10.3 nm, 24.9 nm, and 13.9 nm for the respective mass ratios.

The relationship between resonance wavelength and Ag^+^ concentration, extracted from Figure 4a–c, is depicted in Figure 4d–f. Within the concentration range of 0 to 0.1 μg/L of Ag^+^ solution, the resonance wavelength linearly correlates with the Ag^+^ concentration. However, as the concentration increases from 0.1 μg/L to 1 μg/L, the resonance wavelength shift gradually saturates. Therefore, the linear working range for Ag^+^ detection is established to be 0–0.1 μg/L. This phenomenon can be attributed to the abundant binding sites on the MoS_4_-LDH-sensitive layer at lower Ag^+^ concentrations, resulting in a significant wavelength shift. As the Ag^+^ concentration rises, the binding sites on the sensitive layer become occupied, leading to a reduction in the resonance wavelength shift. Further analysis, detailed in the insets of Figure 4d–f, confirms a high linear correlation coefficient for the fitted linear ranges (with R^2^ being 0.98, 0.99, and 0.95), indicating a strong linear relationship between the resonance wavelength of the sensor and the Ag^+^ concentration within this range. Additionally, this study identifies that at a (NH_4_)_2_MoS_4_ to CO_3_-LDH ratio of 1:8, the SPR sensor achieves a maximum detection sensitivity of 254.75 nm/μg/L. Consequently, the optimal ratio of (NH_4_)_2_MoS_4_ to CO_3_-LDH for subsequent MoS_4_-LDH experiments was determined to be 1:8. This precursor ratio is considered to enhance the availability of binding sites for Ag^+^ adsorption.

**Figure 5:**
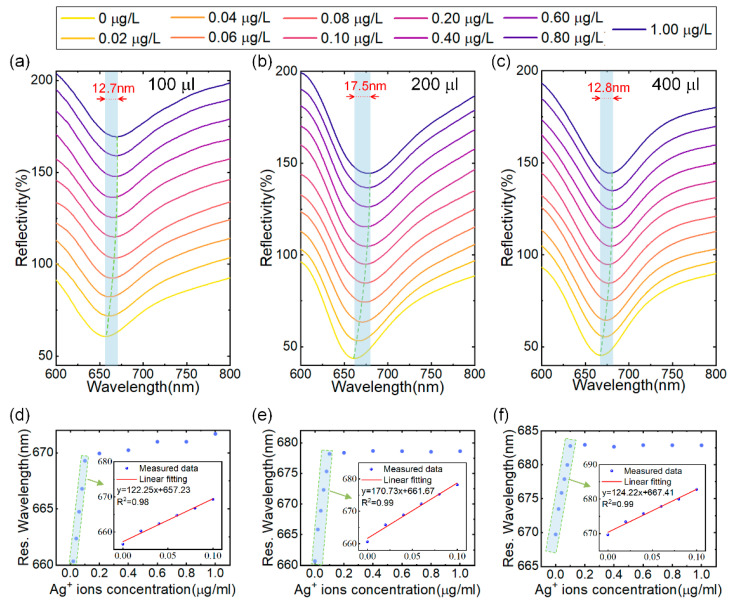
(**a**–**c**) Reflectance spectra of SPR sensors coated with MoS_4_-LDH at precursor ratios at spray volumes of 100 μL, 200 μL, and 400 μL, respectively, in response to varying Ag^+^ ion concentrations. (**d**–**f**) Extracted resonant wavelength shifts as a function of Ag^+^ ion concentrations from (**a**–**c**), with insets showing the linear range fitting.

Next, we investigated the influence of the sensitive layer thickness on sensor performance by varying the amount of MoS_4_-LDH solution sprayed on the surface of the sensor. The concentration of the MoS_4_-LDH dispersion was maintained at 2.5 mg/mL, and SPR sensors were prepared using applied volumes of 100 μL, 200 μL, 300 μL and 400 μL. These sensors were subsequently tested with Ag^+^ solutions across a concentration range of 0 to 0.1 μg/L, as illustrated in Figure 5a–c. (Note: results for the 300 μL volume are already presented in Figure 4.) The resonance wavelength shifts observed were 12.7 nm, 17.5 nm, and 12.8 nm for the sensors with applied volumes of 100 μL, 200 μL, and 400 μL, respectively, as the Ag^+^ concentration increased from 0 to 0.1 μg/L. In Figure 5d,e, the extracted resonance wavelengths were plotted against the Ag^+^ concentration, revealing a red shift that approached saturation with increasing Ag^+^ concentration. The insets in these figures present the linear fit regions, demonstrating a strong linear relationship between the resonance wavelengths and Ag^+^ concentration, with correlation coefficients greater than 0.98 in all cases.

**Figure 6:**
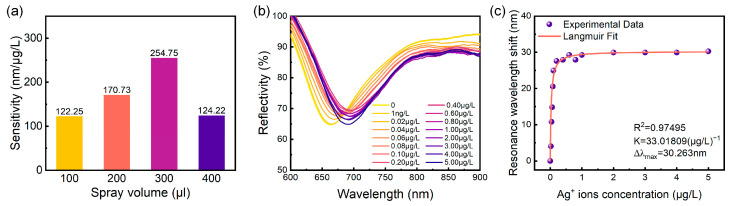
(**a**) Sensitivity as a function of spray volume at precursor ratios of (NH_4_)_2_MoS_4_ to CO_3_-LDH of 1:8. (**b**) Variation in the SPR sensor reflection spectrum with Ag^+^ concentration in the solution, ranging from 0 to 5 μg/L. (**c**) Extracted resonant wavelength versus Ag^+^ concentration from (**b**) along with the Langmuir fit.

Based on the findings presented in Figure 4 and Figure 5, we constructed a graph depicting the relationship between sensor detection sensitivity and the applied volume, as shown in Figure 6a. The results indicate that sensor detection sensitivity initially increases and subsequently decreases with increasing material volume applied to the sensitive layer. The maximum detection sensitivity of 254.75 nm/μg/L was achieved with a material volume of 300 μL. Consequently, the optimal mass ratio of (NH_4_)_2_MoS_4_ to CO_3_-LDH material was determined to be 1:8, with an optimal spray volume of 300 μL.

The spray volume directly influences the thickness of the sensitive layer, as illustrated in Figure 3. A thin MoS_4_-LDH sensitive layer (e.g., spray volume of 100 μL) offers limited binding sites for Ag^+^, hindering high-sensitivity detection. On the other hand, while a thicker MoS_4_-LDH layer (e.g., spray volume of 400 μL) provides abundant binding sites, the silver ions tend to accumulate near the upper surface, away from the evanescent field region of the SPR sensor, thus reducing sensitivity. Therefore, an optimal intermediate layer thickness has been determined, corresponding to a spray volume of 300 μL and a layer thickness of approximately 370 nm (Figure 3c). This configuration provides sufficient binding sites within the evanescent field region, thereby maximizing detection sensitivity.

Subsequent experiments were conducted to evaluate the detection limit of the prepared MoS_4_-LDH-based SPR sensor at the optimal mass ratio and spray volume. To this end, the concentration range of Ag^+^ ion solutions was expanded from 1 μg/L to 5 μg/L, and the spectral response of the SPR sensor was assessed. The experimental results are presented in Figure 6b. Notably, at an Ag^+^ concentration as low as 1 ng/L, the resonance wavelength shift in the reflection spectrum was measured to be 1.026 nm, demonstrating the capability of the sensor to detect trace amounts of Ag^+^ ions down to such a concentration. Within the concentration range of 0 to 0.1 μg/L, the resonance wavelength shift exhibited a linear increase; however, at Ag^+^ concentrations exceeding 0.1 μg/L, the wavelength shift gradually approached saturation. These trends are consistent with the findings presented in Figure 4 and Figure 5.

The resonance wavelength shifts corresponding to various Ag^+^ concentrations, as presented in Figure 6b, were extracted and analyzed using the Langmuir isotherm adsorption model [33], expressed as follows:

(1) $$\Delta \lambda =\Delta \lambda_{\max}\left(\frac{KC}{1+KC}\right)$$

where Δ*λ* is the wavelength shift due to adsorption of the Ag^+^ onto the MoS_4_-LDH sensing layer, Δ*λ*_max_ is the maximum wavelength shift at saturation, *C* is the concentration of Ag^+^ solution, and *K* is the equilibrium binding constant under steady-state conditions. The fitting results, shown in Figure 6c, indicate that the MoS_4_-LDH-based SPR sensor possesses a high binding affinity for Ag^+^ ions, with a binding affinity constant *K* of 33 (μg/L)^−1^ and Δ*λ*_max_ of 30 nm. Additionally, the experimental data show a strong fit with the Langmuir model (R^2^ = 0.98).

The resolution of the spectrometer is the key factor limiting the detection limit of the sensing system, assuming that environmental noise is not dominant. Consequently, by making the value of Δ*λ* in Langmuir isotherm model equation equal to the spectrometer resolution (0.3 nm), the expression of the lowest detection limit *C_LOD_* of the sensor can be obtained, which is expressed by a modified equation as follows:

(2) $$C_{LOD}=\frac{\Delta \lambda }{K\left(\Delta \lambda_{\max}-\Delta \lambda \right)}$$

Substituting the *K* and Δ*λ*_max_ values obtained from the curve fitting using the Langmuir isotherm model into the above equation, the concentration detection limit of the proposed sensor has been calculated to be 0.303 ng/L (2.8 pM), which is lower than previously reported sensors for Ag^+^. Table 1 summarizes the detection limits of the proposed sensor in comparison to previously reported sensors using different methods and materials. It is evident that our SPR sensor exhibits superior performance.

Due to the presence of numerous metal ions and biological macromolecules in real environmental samples, the composition is complex and may affect the detection results of the MoS_4_-LDH-based SPR sensor for Ag^+^ ions. To evaluate the performance of the detector for low-concentration Ag^+^ in real environmental samples, spiking detection was conducted using collected samples from the Yangtze River and tap water in Chongqing. Prior to testing, the river water samples underwent precipitation, filtration, and centrifugation to remove larger solid particles and plankton. Three spectral response tests were performed on different environmental samples, and the recovery rates (the percentage of detected concentration relative to the calibration concentration) of the MoS_4_-LDH-based SPR sensor for Ag^+^ in tap water and river water were calculated. The results, presented in Table 2, showed that the recovery rates of the sensor for Ag^+^ at concentrations of 0.02 μg/L and 0.04 μg/L were 105% and 98.75% in tap water, and 97% and 101.5% in river water, respectively, with relative standard deviations below 4.76%. These findings indicate that the sensor maintains high accuracy for the quantitative determination of Ag^+^ in real environmental water samples with complex and diverse compositions.

**Figure 7:**
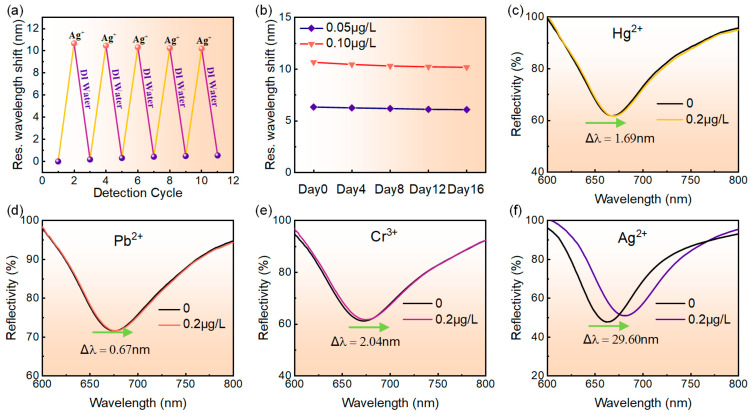
(**a**) Repeatability test results of the sensor. (**b**) Long-term stability assessment of the sensor. (**c**–**f**) Variation in the reflection spectra of the sensor as the concentration of metal ions increases from 0 to 0.2 μg/L, specifically for Hg^2+^, Pb^2+^, Cr^3+^, and Ag^+^, respectively.

The repeatability of the MoS_4_-LDH-based SPR sensor was assessed through five cycles of adsorption and desorption processes. Initially, the reflection spectrum of deionized water was recorded as a reference, followed by testing with a 0.1 μg/L Ag^+^ solution to capture its reflection spectrum. Prior to each subsequent cycle, the MoS_4_-LDH-sensing layer was rinsed with deionized water to remove adsorbed Ag^+^. The resonance wavelength shift of the sensor was measured for both deionized water and the 0.1 μg/L Ag^+^ solution across different cycles. Figure 7a presents the results, showing resonance wavelength shifts of 10.65 nm, 10.44 nm, 10.30 nm, 10.22 nm, and 10.17 nm over the five cycles, with a total decrease of only 0.48 nm. This indicates that the MoS_4_-LDH-based SPR sensor exhibits excellent repeatability.

The long-term stability of the sensor was evaluated by monitoring its response to 0.05 μg/L and 0.1 μg/L Ag^+^ solutions over 16 days. The resonance wavelength shift was recorded, and the relationship between test days and resonance wavelength shift was plotted in Figure 7b. Tests were conducted every three days, with the sensor surface cleaned with deionized water after each test. The results indicated that for the 0.05 μg/L Ag^+^ solution, the resonance wavelength shift decreased from 6.33 nm to 6.08 nm, representing only a 3.9% reduction from the initial measurement. For the 0.1 μg/L Ag^+^ solution, the shift decreased from 10.65 nm to 10.17 nm, a mere 4.5% reduction. These findings demonstrate the good long-term stability of the sensor.

The specific detection capability of the MoS_4_-LDH-based SPR sensor for Ag^+^ was evaluated. Four standard solutions of heavy metal ions, including Hg^2+^, Pb^2+^, Cr^3+^ and Ag^+^, were prepared at concentrations of 0.2 μg/L each for testing. The responses of the SPR sensor to these different heavy metal ions were compared and analyzed. Figure 7c–e display the reflection spectra of the SPR sensor for detecting the four heavy metal ions. The figure reveals that as the concentration of the ion solution increases from 0 to 0.2 μg/L, the resonance wavelength shifts of the reflection spectra are 1.69 nm, 0.67 nm, 2.04 nm, and 29.60 nm, respectively. Clearly, under the same concentration of heavy metal ion solution, the SPR sensor designed for detecting Ag^+^ exhibited the largest resonance wavelength shift, whereas the shifts for detecting Hg^2+^, Pb^2+^, and Cr^3+^ were only 5.72%, 2.27%, and 6.89%, respectively, of that for detecting Ag^+^. These results indicate that the SPR sensor exhibits high selectivity for Ag^+^ and is suitable for the specific detection of low concentrations of Ag^+^.

For a clearer understanding of our findings, we assessed the selectivity of CO_3_-LDH-based SPR sensors for comparative analysis. The results indicate that the resonant shifts of these sensors in response to solutions containing Ag^+^, Hg^2+^, Pb^2+^, and Cr^3+^ are in the same order, suggesting that these sensors exhibit limited selectivity for Ag^+^ ions. Consequently, these findings underscore the significant role of the MoS_4_^2−^ anion in the adsorption of Ag^+^. Indeed, previous studies have shown that the MoS_4_^2−^ anion exhibits the highest selectivity for Ag^+^ [38,39,40], in accordance with our experiment’s results. Regarding the adsorption mechanisms of Ag^+^ by MoS_4_-LDHs, two possible mechanisms may be predominant. First, interlayer chelation is likely the primary mechanism, where the MoS_4_^2−^ anion binds to Ag^+^ to form an Ag_2_[MoS_4_] complex. Additionally, a portion of the Ag^+^ could also be adsorbed onto the hydroxide-containing layers of the LDHs, as demonstrated previously [39,40].

At last, we discuss the limitations of the SPR sensor. Firstly, the performance of the sensor may be compromised by interference from non-target substances in real environmental samples, such as macromolecules and particulates. These substances, when adsorbed onto the LDHs layer, can modify the refractive index of the material, underscoring the need for sample purification. Secondly, there remains potential for further improvement in the linear measurement range of the sensor. Balancing a broad linear range with high sensitivity is challenging due to the finite number of binding sites on the material. Typically, expanding the linear range requires lowering the binding affinity, which can decrease sensitivity, while increasing sensitivity by enhancing binding affinity tends to narrow the linear range. To overcome this challenge, future research could explore increasing the number of adsorption sites for Ag^+^, possibly through innovative functionalization of LDHs or other new materials.

## 5. Conclusions

In summary, this paper presents a novel label-free SPR sensor for Ag^+^ detection with enhanced sensitivity and selectivity. The sensor incorporates MoS_4_^2−^-intercalated NiAl-LDH nanosheets as the sensitive layer, where MoS_4_^2−^ anions replace the original CO_3_^2−^ anions in the LDH structure, resulting in a material with high adsorption capacity and strong selectivity for Ag^+^. Characterization techniques, including TEM, XPS, XRD and FTIR, confirmed the successful synthesis of the MoS_4_-LDH nanosheets. By optimizing the precursor ratio of MoS_4_-LDH and spray volume on the sensor surface, the sensor achieved a remarkable sensitivity of 254.75 nm/μg/L for Ag^+^. Analysis using the Langmuir adsorption model revealed an exceptionally low detection limit of 2.8 pM, surpassing various previously reported Ag^+^ sensors based on different detection methods and materials. The sensor demonstrated excellent repeatability, long-term stability, and high selectivity for Ag^+^. Additionally, the sensor exhibited a recovery rate of Ag^+^ between 95% and 105% in tap and river water samples, indicating its utility in real-world environmental applications. This work introduces a promising sensor for highly sensitive Ag^+^ detection, paving the way for the development of high-performance SPR sensors optimized for heavy metal ion detection.

## Figures and Tables

**Table 1:** Comparison of detection limits of several detection methods for Ag^+^.

Material	Method	Detection Limit	**Reference**
BSA-Au nanoclusters	Fluorescence	0.226 μM	[34]
Gold interdigitated microband electrode	Electrochemistry	13 nM	[35]
Carbon dots with dual excitation	Fluorescence probe	15 nM	[36]
Chitosan-AuNPs	Colorimetry	0.13 μM	[37]
MoS_4_-LDH	SPR	2.8 pM	This work

**Table 2:** Recovery rate of Ag^+^ in actual samples of MoS_4_-LDH-based SPR sensor.

Test Environment	Spiked Ag^+^ (μg/L)	Found Ag^+^ (μg/L)	Recovery (*n* = 3)	RSDs (*n* = 3)
Tap water	0.02	0.0210	105%	4.76%
	0.04	0.0395	98.75%	2.28%
River water	0.02	0.0194	97%	3.09%
	0.04	0.0406	101.5%	1.94%

## Data Availability

Data underlying the results presented in this paper are not publicly available at this time but maybe obtained from the authors upon reasonable request.
